# Right iliac vein thrombosis mimicking acute appendicitis in pregnancy: a case report

**DOI:** 10.1186/s13104-016-2351-5

**Published:** 2017-01-03

**Authors:** Desmond Aroke, Benjamin Momo Kadia, Christian Akem Dimala, Ndemazie Nkafu Bechem, Larry Tangie Ngek, Simeon Pierre Choukem

**Affiliations:** 1 Nkwen Baptist Health Center, Bamenda, Cameroon; 2 Health and Human Development (2HD) Research Group, Douala, Cameroon; 3 Presbyterian General Hospital Acha-Tugi, Acha, Cameroon; 4 Faculty of Epidemiology and Population Health, London School of Hygiene and Tropical Medicine, London, UK; 5 Penka Michel District Hospital, Penka-Michel, Cameroon; 6 Banso Baptist Hospital, Kumbo, Cameroon; 7 Faculty of Health Sciences, University of Buea, Buea, Cameroon; 8 Department of Internal Medicine, Douala General Hospital, Douala, Cameroon

**Keywords:** Right iliac vein thrombosis, Pregnancy, Appendicitis, Case report

## Abstract

**Background:**

Right iliac vein thrombosis is uncommon in pregnancy. Nonetheless, when it does occur, its presentation could be very unspecific with important diagnostic challenges and this could have negative therapeutic consequences especially in a resource limited setting.

**Case presentation:**

The historical, clinical and laboratory data of a 30 year old G2P1001 woman of African ethnicity at 11 weeks of gestation pointed towards a right iliac vein thrombosis missed for an acute appendicitis with subsequent appendectomy and failure to cure. Following the diagnosis of right iliac vein thrombosis post-appendectomy, the patient was started on low molecular weight heparin and the clinical progress thereafter was favourable.

**Conclusion:**

Pelvic vein thrombosis should be considered a differential diagnosis of intractable lower abdominal pain in early pregnancy. A high index of suspicion could lead to early diagnosis, prompt management and a favourable prognosis even in a low-income setting.

## Background

Pregnancy is a hypercoagulable state, during which the risk of both arterial and venous thromboembolism is increased although venous thromboembolism (VTE) predominates [1]. This hypercoagulable state results from a combination of altered coagulation factors, stasis and vascular damage [1]. VTE is a well known cause of maternal mortality worldwide and is the leading cause of maternal mortality in the United Kingdom [2]. However there is not much data on the burden of VTE amongst pregnant women in low and middle income countries. Deep venous thrombosis (DVT) accounts for about 85% of cases of VTE in pregnancy [3], with 2/3 of these cases shown to occur during the antepartum period [4]. DVT in pregnancy is more likely to be proximal with about 90% of cases occurring in the right side. Pelvic vein thrombosis (PVT) is uncommon outside pregnancy but accounts for 10% of pregnancy-related DVT [1]. PVT presents with symptoms similar to those of acute appendicitis such as; fever, abdominal pains, nausea and vomiting [5–7]. This could raise serious diagnostic and management dilemmas with negative therapeutic consequences; especially in resource-poor settings where the appropriate diagnostic and management arsenal is not always available.

We report the case of a patient with a non specific picture of PVT missed for and managed as acute appendicitis with subsequent development of severe bilateral lower limb DVT in a 1st trimester pregnancy.

## Case presentation

A 30 year old G2P1001 sub-Saharan African female teacher at 11 weeks amenorrhea, presented to the Nkwen Baptist Health Center (Bamenda, North West Region of Cameroon) on the 15th of May 2016 with bilateral lower limb swelling and pain of 5 days duration. She had no known chronic illness and denied having a family history of VTE.

She reported being well till 2 weeks prior to presentation when she started experiencing abdominal pains; the pain was mainly in her lower abdomen, dull in nature, non-radiating, mild in intensity, was initially intermittent then became constant. It was associated with intermittent low grade fever. This prompted her to consult at a remote health center, where a urinalysis and malaria parasite test was done but their results were inconclusive. She was then cautioned to be having early pregnancy symptoms and placed on acetaminophen 3 g per day in three divided doses which she took for a week with no regression of symptoms. The persistent and progressively worsening pain now localized at the right lower quadrant prompted a second consultation at another health facility. This pain was still associated with low grade fever and now included; loss of appetite and intermittent postprandial vomiting. The attending physician on examination remarked right iliac fossa tenderness and rebound tenderness with a positive Rovsing’s sign. Presumptive diagnosis of acute appendicitis and differential of ovarian cyst in pregnancy were retained. An emergency surgery was booked. However, intra-operative findings revealed a normal appendix and ovaries.

Following surgery, lower abdominal pains persisted and she complained of a sudden onset of crampy constant pains in her right thigh. She was told to be having post surgery pain, for which she was then given analgesics. On day 3 post hospitalization she was discharged on analgesics, antibiotics and progesterone suppository. While at home, the pains persisted and 2 days later involved her left calf area. This was associated with bilateral lower limb swelling that was more on the right lower limb. The pain increased in severity making it difficult for her to walk. This prompted consultation at our health facility.

On arrival she was ill-looking and in painful distress. Her blood pressure was 122/76 mmHg, heart rate 94 beats/min, respiratory rate of 22 breaths/min, temperature 37 °C, O_2_ saturation at 97% and weight 58 kg. Her conjunctivae were pink and sclera anicteric, heart sounds were normal and lung fields clear. On examining the abdomen, a clean midline incision was seen and there was tenderness on deep palpation of the lower abdominal quadrants marked on the right. There was bilateral lower limb pitting oedema extending to the thighs with right lower limb more swollen than left. The limbs were mildly erythematous but there was no area of cracked skin or wound on both limbs that could have served as portal of entry for skin infection. Both lower limbs were warm tender.

Based on these we made a tentative diagnosis of bilateral lower limb deep venous thrombosis in early pregnancy with a possible pelvic vein thrombosis that was misdiagnosed for acute appendicitis. Our health facility was not equipped with the necessary tools and personnel to confirm our diagnosis and manage the patient. She was therefore referred to a tertiary care center about 40 km from our facility.

**Fig. 1:**
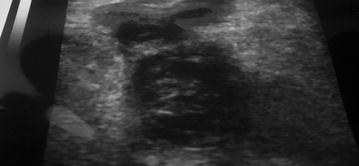
Echography showing the thrombus present as echoic image inside the right common iliac vein

At the tertiary center compressive doppler ultrasound of the pelvis and lower limbs revealed pelvic and bilateral lower extremity veins seen with echoes in the right common iliac vein (Fig. 1), right femoral vein, left femoral vein and left popliteal vein. There was decreased colour flow in these veins and decreased compressibility. These suggested DVT of the right common iliac vein, right femoral vein, left femoral vein and left popliteal vein and thus confirmed our diagnosis of bilateral lower limb and pelvic DVT.

Further laboratory testing showed the following: normal white cell count of 8100/µl, mild anaemia with haemoglobin of 9.8 g/dl, thrombocytosis of 532,000/µl, normal kidney function test (serum creatinine of 0.64 mg/dl and urea of 12.7 mg/dl), glycaemia of 85.9 mg/dl and normal serum electrolytes of: (Sodium 134 mmol/l, Potassium of 4.17 mmol/l and Chloride of 103 mmol/l). Cardiac echography and electrocardiogram done were all normal.

**Fig. 2:**
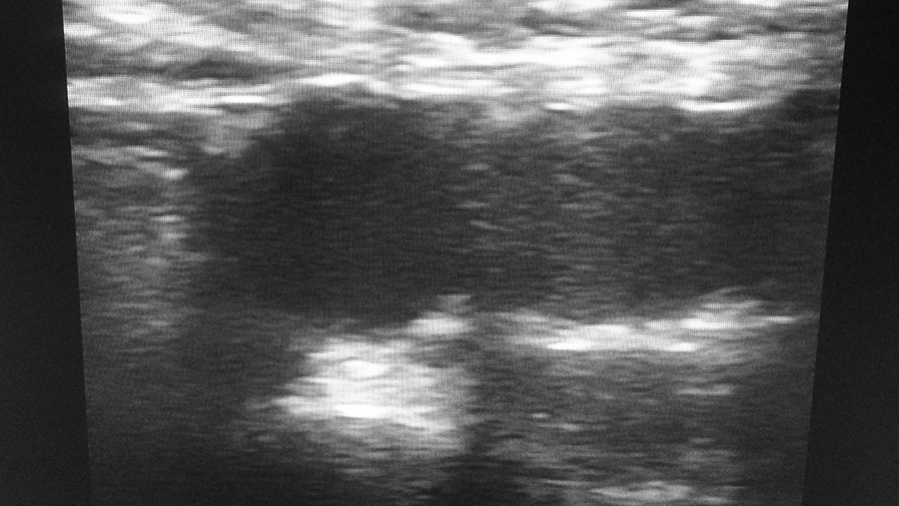
Echography showing no more echoic images inside the right common iliac vein

The patient was immediately started on low molecular weight heparin (LMWH) 80 mg subcutaneous route daily. After 5 days of treatment the patient’s symptoms had subsided and she was discharge and counter referred for continuation of care. We continued her daily LMWH injections and scheduled her for a repeat of the pelvic and lower limb ultrasound. Six weeks later there were no more echoes in the pelvic and lower limb veins (Fig. 2). She continued daily LMWH till 12 weeks postpartum.

## Conclusion

Pregnancy generally increases the risk of VTE by fivefolds [8]. Unlike in the developed world where VTE in pregnancy is the most common cause of maternal mortality [9], postpartum haemorrhage and preeclampsia are the most common causes of maternal mortality in developing countries. VTEs are thus not routinely considered as a priority differential in most African countries by physicians when approaching pregnant women with acute abdominal pain. Consequently, VTEs in pregnancy in low and middle income countries are more likely to be missed [10]. The epidemiological and clinical burdens of VTEs in pregnancy in the African context may be grossly underestimated because of under/misdiagnosis and possibly an associated high case fatality rate particularly in sub-urban areas. A particularity in the case presented is that though pregnancy increases risk of DVT, its occurrence in the 1st trimester of pregnancy is relatively low [4]. Thus, making a diagnosis of PVT especially in early pregnancy will require a high index of suspicion.

Pelvic vein thrombosis is uncommon outside pregnancy and accounts for 10% of pregnancy related DVTs [8]. Despite this, studies have shown that approximate 60% of proximal DVTs are limited to the femoral and iliac veins [11]. PVT may thus be more frequent than suspected. PVT typically presents with symptoms similar to those of appendicitis such as; fever, abdominal pains, nausea and vomiting [5, 6]. These two conditions could therefore often be confused when judging only by clinical presentation.

The clinical presentation of PVT is similar to that of acute appendicitis. The latter being a frequent cause of acute abdominal pain, is a notoriously preferred aetiological diagnosis in most cases of right iliac fossa pain especially in poor settings. Furthermore, it is difficult to differentiate between PVT and other pelvic pathologies such as torsion of ovarian cyst, pelvic inflammatory diseases, ureteral colic, enteritis or an ectopic pregnancy solely on clinical basis. Therefore clinical vigilance and the ability to rule out other differential diagnoses are essential as untreated DVT can have devastating consequences and could even be lethal.

Pulmonary embolism (PE) is a well known fatal complication of DVT. Placement of an inferior vena cava filter is usually necessary in preventing this complication in patients who are not candidates for anticoagulation or who have had a previous PE while on therapeutic anticoagulants [12]. However, our patient did not have features suggestive of a PE. Early diagnosis of DVT is therefore necessary for prompt treatment and to prevent development of these complications and sequela. Diagnosis of DVT can be made using ultrasonography, computed tomography, magnetic resonance imaging or venography. Magnetic venography is the method of choice for the diagnosis of PVT [1]. In resource poor settings where these equipments are not readily available, diagnosis is usually done by ultrasound as exemplified in the case presented. However, studies have shown that in pregnancy there is a fair agreement between ultrasound and magnetic resonance imaging for determination of the extend of DVT into pelvic veins [13].

Pelvic vein thrombosis is usually managed conservatively with anticoagulant therapy and in rare cases surgically. Favourable outcome with aggressive surgical therapy has been reported in a 35 weeks pregnancy with iliofemoral-popliteal DVT in Germany [14]. Intravenous antibiotics can also be used in case of sepsis. Treatment in pregnancy requires consideration of the twin issues of safety for the fetus and the mother. The standard treatment for VTE outside of pregnancy is LMWH in the acute phase for at least 5 days associated with warfarin, which is then continued for 3–6 months [15]. Warfarin is avoided in pregnancy as it crosses the placenta [9]. LMWH is at least as effective and safe as un-fractionated heparin for treatment of VTE and the effects more predictable with no requirement for routine monitoring [16]. In this case there was no evidence of infection so the patient was treated conservatively with daily LMWH injections and the outcome thereafter was favourable.

The case presented confirms that PVT has a similar presentation to and is often confounded with acute appendicitis. PVT should thus be considered in any pregnant woman presenting with unexplained lower abdominal pains with a clinical picture similar to that of acute appendicitis. Clinicians should have a high index of suspicion in order to make a proper and early diagnosis so as to optimize the prognosis.

## Abbreviations

VTEs: venous thromboembolism
DVT: deep venous thrombosis
RIVT: right iliac vein thrombosis
PVT: pelvic vein thrombosis
LMWH: low molecular weight heparin
PE: pulmonary emboli

## References

[1] Arya R (2011). How I manage venous thromboembolism in pregnancy. Br J Hematol.

[2] Centre for Maternal and Child Enquiries (CMACE) (2011). Saving mothers’ lives: reviewing maternal deaths to make motherhood safer: 2006–08. The eighth report on confidential enquiries into maternal deaths in the United Kingdom. Br J Obstet Gynaecol.

[3] James AH, Tapson VF, Goldhaber SZ (2005). Thrombosis during pregnancy and the postpartum period. Am J Obstet Gynecol..

[4] Ray JG, Chan WS (1999). Deep vein thrombosis during pregnancy and the puerperium: a meta-analysis of the period of risk and the leg of presentation. Obst Gynecol Surv.

[5] Björgell O, Nilsson P, Nilsson A, Lorén I, Florén C, Lindgärde F (1998). Isolated internal iliac vein thrombosis. J Ultrasound Med.

[6] Merhi ZAA (2006). Acute abdominal pain as a the presenting symptom of isolated iliac vein thrombosis in pregnancy. Obstet Gynecol.

[7] Visaria SDDJ (2002). Pelvic vein thrombosis as a cause of acute pelvic pain. Obstet Gynecol.

[8] Heit JA, Kobbervig CE, James AH, Petterson TM, Bailey KRMLI (2005). Trends in the incidence of venous thromboembolism during pregnancy or postpartum: a 30-year population- based study. Ann Intern Med.

[9] Bates SM, Greer IA, Pabinger I, Sofaer SHJ (2008). Venous thromboembolism, thrombophilia, antithrombotic therapy, and pregnancy. American college of chest physicians evidence-based clinical practice guidelines. Chest.

[10] Jose Manuel Ceresetto (2016). Venous thromboembolism in Latin America: a review and guide to diagnosis and treatment for primary care. Clinics..

[11] Chan WS, Spencer FAGJ (2010). Anatomic distribution of deep vein thrombosis in pregnancy. CMAJ.

[12] Suleyman T, Gultekin H, Abdulkadir G, Tevfik P, Abdulkerim UM, Ali A, Ismail K (2008). Acute right lower quadrant abdominal pain as the presenting symptom of ovarian vein thrombosis in pregnancy. J Obstet Gynaecol Res..

[13] Torkzad MR, Bremme K, Hellgren M, Eriksson MJ, Hagman A, Jorgensen T, Lund K, Sandgren G, Blomqvist L, Kälebo P (2010). Magnetic resonance imaging and ultrasonography in diagnosis of pelvic vein thrombosis during pregnancy. Thromb Res.

[14] Burgazli KM, Hakan A, Bilgin M, Kavukcu E, Päfgen W, Ertan AK (2012). Iliofemoral-popliteal deep vein thrombosis at 35th week of pregnancy : treated with cesarean section and vena cava blockage plus thrombectomy. J Turkish-German Gynecol Assoc..

[15] Kearon C, Kahn SR, Agnelli G, Goldhaber S, Raskob GE, Comerota AJ. Antithrombotic therapy for venous thromboembolic disease. American college of chest physicians evidence-based clinical practice guidelines. 8th ed. 2008. p. 454S–545S. 10.1378/chest.08-0658 18574272

[16] Gould MK, Dembitzer AD, Doyle RL, Hastie TJ, AM Garber (1999). Low-molecular weight heparins compared with unfractionated heparin for treatment of acute deep venous thrombosis. A meta-analysis of randomized, controlled trials. Ann Intern Med.

